# Caspase 5 depletion is linked to hyper-inflammatory response and progeroid syndrome

**DOI:** 10.1007/s11357-023-00907-1

**Published:** 2023-08-21

**Authors:** Fuki M. Hisama, Renuka Kandhaya Pillai, Julia Sidorova, Karynne Patterson, Carolina Gokingco, Michal Yacobi-Bach, Junko Oshima

**Affiliations:** 1 Department of Medicine, Division of Medical Genetics, University of Washington, Seattle, USA; 2 Department of Laboratory Medicine and Pathology, University of Washington, Box35747, Seattle, 98195 USA; 3 Department of Genome Science, University of Washington, Seattle, USA; 4 Endocrine and Genetics Institutes, Sourasky Medical Center, Tel Aviv, Israel

**Keywords:** CASP5, Inflammation, Progeroid syndrome, Aging, Human, Genetic disorder

## Abstract

A progeroid family was found to harbor a pathogenic variant in the *CASP5* gene that encodes inflammatory caspase 5. Caspase 5-depleted fibroblasts exhibited hyper-activation of inflammatory cytokines in response to pro-inflammatory stimuli. Long-term intermittent hyper-inflammatory response is likely the cause of the accelerated aging phenotype comprised of earlier onset of common aging diseases, supporting inflammaging as a potential common disease mechanism of progeroid syndromes and possibly normative aging.

Segmental progeroid syndromes are groups of disorders that present with features of accelerated aging affecting multiple organs and tissues and have provided novel biological insights into cellular mechanisms of aging. Prototypical examples are adult-onset Werner syndrome caused by loss of *WRN* helicase function [17] and child-onset Hutchinson-Gilford progeria syndrome caused by abnormal nuclear lamin, termed progerin [4]. During the past decades, the International Registry of Werner Syndrome (Seattle, USA) identified progeroid loci that highlight major roles in DNA damage repair and response: *WRN* (RecQ helicase) [17], *LMNA* (nuclear structure and chromatin interaction) [2], *POLD1* (DNA polymerase delta) [10], *SPRTN* (recruitment of translational DNA polymerase eta) [9], *ERCC4* (nucleotide excision repair) [13], *MDM2* (an inhibitor of p53) [11], *CTC1* (telomere replication) [16], and *SAMHD1* (regulation of dNTP pools) [8]. We and other investigators have observed DNA damage, accelerated telomere shortening, upregulation of p53, enhanced rates of somatic mutation, and mitochondrial dysfunction as potential common mechanisms of accelerated aging [12].

An Israel progeroid pedigree was referred to the Werner Registry. At the time of referral, the index individual (II:3, Registry# TL1010) is a 54-year-old Israeli female with osteoporosis, bilateral cataracts diagnosed at age 47, premature graying of hair since age 12, short stature, thin limb, pinched facial features, high-pitched hoarse voice, calcinosis cutis, and evidence of osteoporosis (Fig. 1a, b). Parents are second cousins (Fig.1b, I:1 and I:2). This satisfied the clinical diagnostic criteria of Werner syndrome [14]. The younger brother, II:4, at age 51 shared similar signs. The parents and two siblings, II:1 at age 59 and II:2 at age 59, were unaffected.

**Fig. 1:**
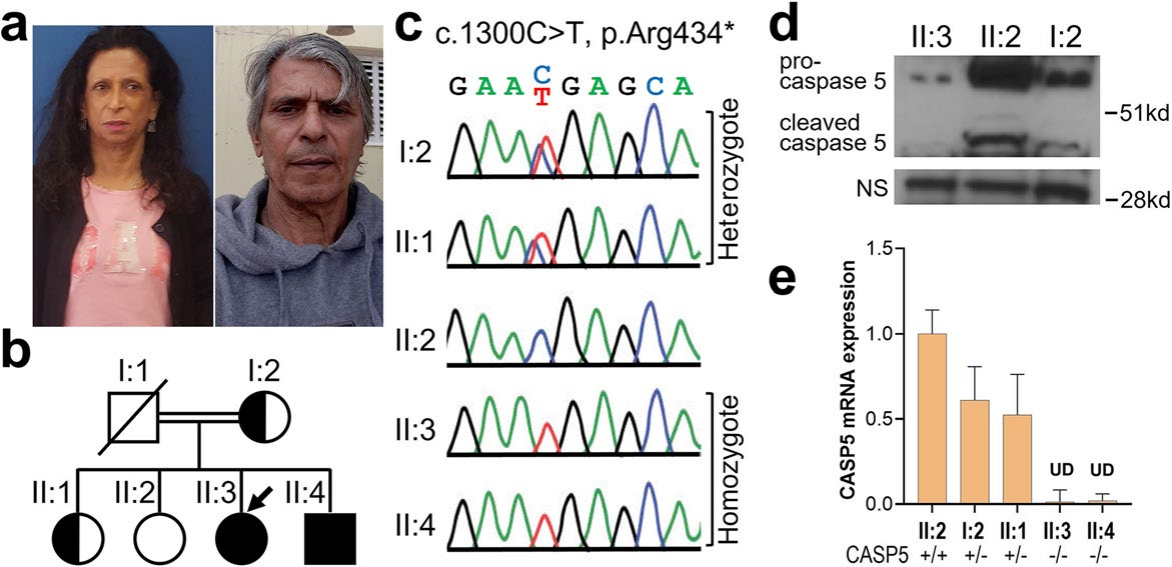
*CASP5* variant and expression in a progeroid pedigree. **a** Proband II:3 at age 54 and the affected brother II:4 at age 51. Patients provided written consent to use these photographs. **b** Family pedigree with the proband (  ), affected (•■), and carriers (  ). **c** *CASP5* sequencing showing the familial variant. **d** Western analysis of caspase 5 in individuals with 3 genotypes. The nonspecific band is shown as a reference. **e** qRT-PCR analysis of *CASP5* mRNA in the same individuals as in **c**

Sanger sequencing of *WRN* exons was negative for pathogenic variants and Western analysis demonstrated *WRN* protein of the expected normal size and quantity (data not shown). Exome sequencing of the index case II:3 revealed a homozygous stop gain, NM_001136112.3: c.1300C>T, p.Arg434*, in exon 9 of the *CASP5* gene. The affected brother II:4 was also homozygous for the variant, and the mother I:2 was heterozygous. We were unable to obtain the sample of the deceased father I:1 who was the obligate heterozygote. One of the unaffected sisters II:1 was heterozygous, and the other unaffected sister II:2 did not carry this alteration. Based on the haplotype, II:1 inherited the *CASP5* c.1300C>T, p.Arg434* allele from the mother (Fig.1b, c). This established the co-segregation within this family.

Western analysis of the lymphoblastoid cell lines (LCLs) showed marked reduction of the *CASP5* gene product, caspase 5 protein in the affected individual II:3 and approximately 50% reduction of caspase 5 protein in the heterozygous individual II:1 relative to the normal individual II:2 (Fig. 2d). The qRT-PCR of *CASP5* showed results similar to the Western blotting, indicating that decline of *CASP5* expression is at mRNA level, likely due to the non-sense mediated decay (Fig. 2b). p.Arg434* is expected to cause 11 amino acid truncation at the C-terminal end of the protein. Although there is no known functional domain in the deleted region, further investigation is needed to evaluate the effect of the trace of the mutant protein.

**Fig. 2:**
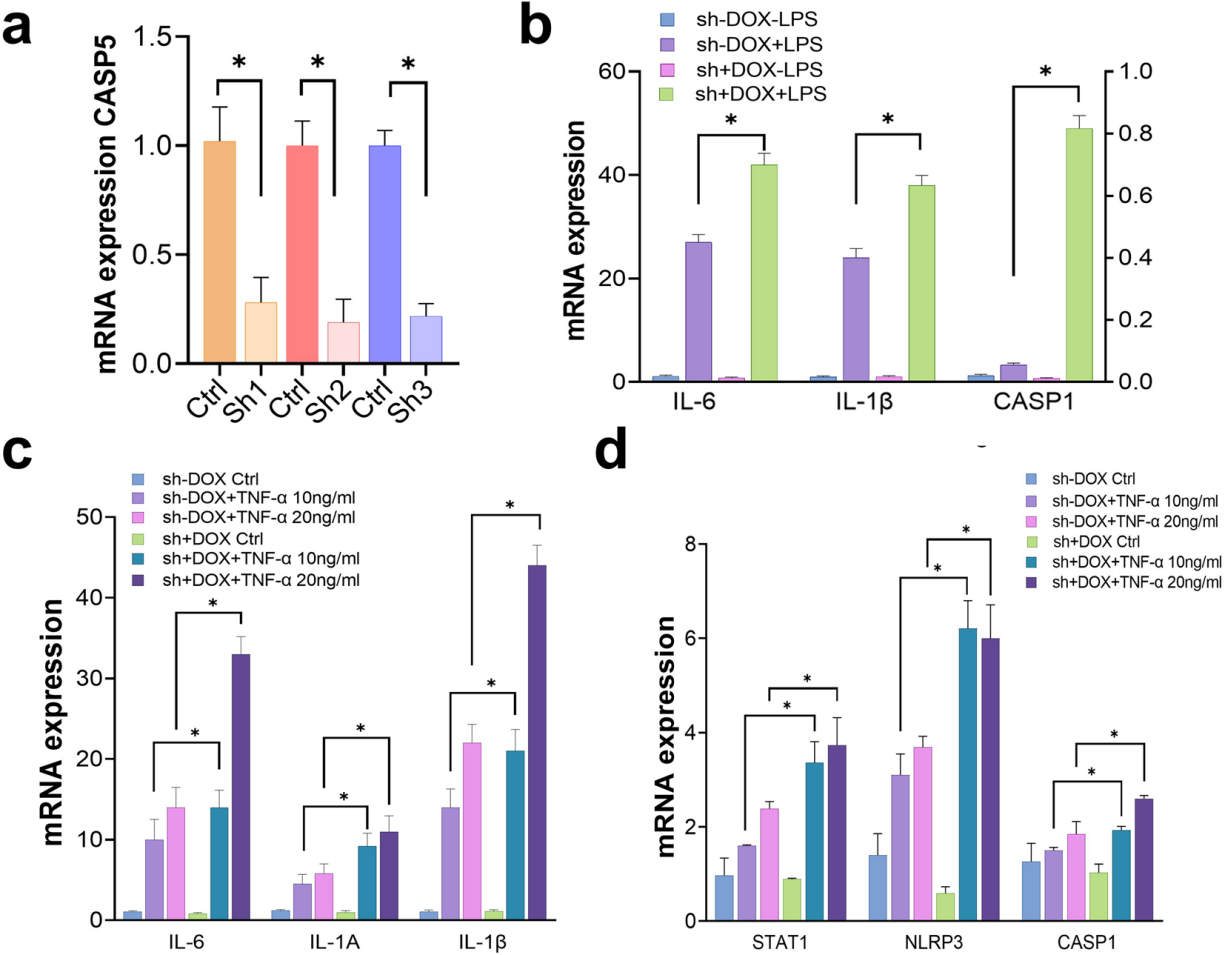
Elevated inflammatory response in CASP5 knockdown fibroblasts. **a** qRT-PCR of *CASP5* in cells with 3 different doxycycline-inducible shCASP5 (Sh1, Sh2, and Sh3), normalized to GAPDH, are shown relative to non-induced culture (Ctrl). * indicates statistical significance. **b** Cytokine induction in Sh3 culture in response to 24-h exposure of 1 μg/ml lipopolysaccharide (LPS), normalized with GAPDH mRNA. **c**, **d** Cytokine induction in Sh3 culture in response to 24-h exposure to TNF-α, 10 ng/ml or 20 ng/ml, normalized with GAPH mRNA. Sh1 and Sh2 cultures showed similar trends (data not shown)

The *CASP5* gene encodes a member of a protease family, caspase 5 [1, 3]. Caspase 5 is known to be involved in the activation of inflammation. We examined the inflammatory cytokines in the LCLs derived from this pedigree. We did not, however, observe consistent changes of cytokines among different *CASP5* genotypes, possibly due to the secondary effect of Epstein-Barr virus transformation as well as individual or clonal differences among the LCLs (data not shown). In order to obtain the isogenic model, we established *CASP5* knockdown fibroblasts using doxycycline-inducible lentiviral shRNAs against *CASP5* [18]. Three *CASP5* shRNA were tested all of which showed nominal decreases of *CASP5* expression as assessed by q-PCR (Fig. 2a).

When *CASP5* knocked down fibroblasts were further treated with pro-inflammatory stimulus, lipopolysaccharide (LPS), IL-6, and IL-1β were induced approximately 60% more in *CASP5*-knocked down fibroblasts, compared to the control cultures (Fig. 2a). Another pro-inflammatory agent, TNF-α, also resulted in approximately 2-fold higher induction of inflammatory factors, (IL-6, IL-1A, and IL-1β), increased expression of STAT1and inflammasome components (NLRP3, and CASP1) in *CASP5* compared to the control culture in dose-dependent manner (Fig. 2c, d). It is conceivable that the absence of caspase 5 may have little effect on inflammatory response under baseline, unstressed conditions, but the presence of pro-inflammatory stimuli could result in the transient hyperactivation of the inflammatory response. In addition, upregulation of caspase 1 reflects inflammasome activation and increased inflammation, which can, in turn, lead to a self-sustaining/positive inflammatory feedback cascade. Our recent study showed that both dysregulated inflammasome and continual secretion of inflammatory cytokines can further amplify the inflammatory response and contribute to hyperinflammatory phenotype [7].

In our previous observation, a progeroid patient with a heterozygous p.Arg496Cys variant of the *SMAD4* was associated with increased senescent makers and the accumulation of DNA damage [6]. The *SMAD4* gene is known to regulate the signaling pathway of TGF-β, a member of SASP. To our knowledge, CASP5-associated accelerated syndrome has not been previously reported. Increased inflammatory response and genomic instability were also seen in the classical Werner syndrome [6]. In the caspase 5-depleted fibroblasts, we did not observe evidence of accumulation of DNA damage in response to LPS or TNF-α as assessed by p53 induction or 53BP1 or γH2AX double strand damage foci (data not shown). This, however, does not exclude the possibility of a transient increase of DNA damage whose detection is dependent on exact timing and conditions. Another possibility is that chronic inflammation might cause accelerated aging without an apparent increase of DNA damage.

We propose that an intermittent or chronic hyper-inflammatory response may be among a suite of common disease mechanisms of progeroid syndromes. The delayed onset of signs of premature aging compared to Werner syndrome (e.g., cataracts in their 40s in *CASP5* homozygotes vs cataracts in their 30s in classical Werner patients [14]) is consistent with the mild but significant increase of inflammatory cytokines.

Imura and colleagues proposed subdividing patients meeting clinical diagnostic criteria for Werner syndrome [5]. They inferred there were at least three distinct clinical types of the disease, with type 1 comprising classical Werner syndrome with pathogenic *WRN* variants and type 2 group having an earlier age of onset, resembling the *LMNA* mutant progeroid syndrome [2]. Type 3 group with late-onset symptoms appears to resemble inflammatory-type progeroid syndrome [5]. The relationship with other markers of aging remains to be determined.

## Methods

### Patient recruitment

The proband was referred to the International Registry of Werner Syndrome (http://www.wernersyndrome.org) for molecular diagnosis of their progeroid syndrome. Prior to the initiation of the study, written informed consent was given by all participants. Patients also provided written informed consent to publish their images. The study complied with the ethical rules specified in the Declaration of Helsinki. This study is approved by the University of Washington Institutional Review Board (ID# STUDY00000233).

### Exome sequencing and analysis

A library of DNA fragments was constructed and enriched for protein and RNA coding portions of the human genome using the Exome v1.0 (Integrated DNA Technologies) capture system. Paired-end sequencing of the enriched library was performed using rapid run v2.0 (Illumina) chemistry on a HiSeq 2500 (Illumina) sequencer according to the manufacturer’s recommended protocol. The resulting sequences were aligned to the human genome reference (hg19) using the Burrows-Wheeler Aligner (BWA) and variants identified with the Genome Analysis Tool Kit (GATK). Variants were initially annotated using an in-house software tool based on SnpEff and subsequently reanalyzed with VEP and an analysis tool, Seqr [6, 13].

### Cell culture

82-6 is a primary human foreskin fibroblast line derived from a newborn. The 88-1pBlox line was generated by retroviral infection of 82-6 with excisable hTERT, pBlox-TSH, followed by histidinol selection [15]. Commercially obtained lentiviral CASP5 shRNAs were introduced to 82-6pBlox following the manufacturer’s instructions: shCASP5-1, V3IHSHER_6488699 (Sh1), V3IHSHER_5488601 (Sh2), and V3IHSHER_10156220 (Sh3) (Horizon Discovery [6, 18]). To induce shRNA expression, 1 μM doxycycline was added to the cultural medium for 5 days prior to the assays [18]. Cultures were maintained under standard culture conditions at 37 °C in an atmosphere of 5% CO2 and 5% O2 [6, 18].

### Western blotting and qRT-PCR analyses

Western blotting and quantitative RT-PCR were performed as previously described [6, 18]. Western blotting analysis utilized commercial antibodies: anti-CASP5 antibody (1:1000, clone D3G4W, #46680, Cell Signaling), anti-β-actin (1:4000, #A1978; Sigma), biotinylated anti-mouse IgG antibody (#BA-9200, Vector Laboratories), and biotinylated anti-rabbit IgG antibody (#BA-1000, Vector Laboratories).

## Data Availability

Data is available from the corresponding author upon request.
